# Supplementary material for: Hsp90 shapes protein and RNA evolution to balance trade-offs between protein stability and aggregation
Source: Nat Commun. 2018 May 3;9:1781. doi: 10.1038/s41467-018-04203-x (PMC5934419; doi:10.1038/s41467-018-04203-x)
Supplement: Supplementary file 1 — Supplementary Information [file 41467_2018_4203_MOESM1_ESM.pdf]

Supplementary Information for

Hsp90 shapes protein and RNA evolution to balance trade-offs between protein stability and aggregation

Geller, Pechmann et al

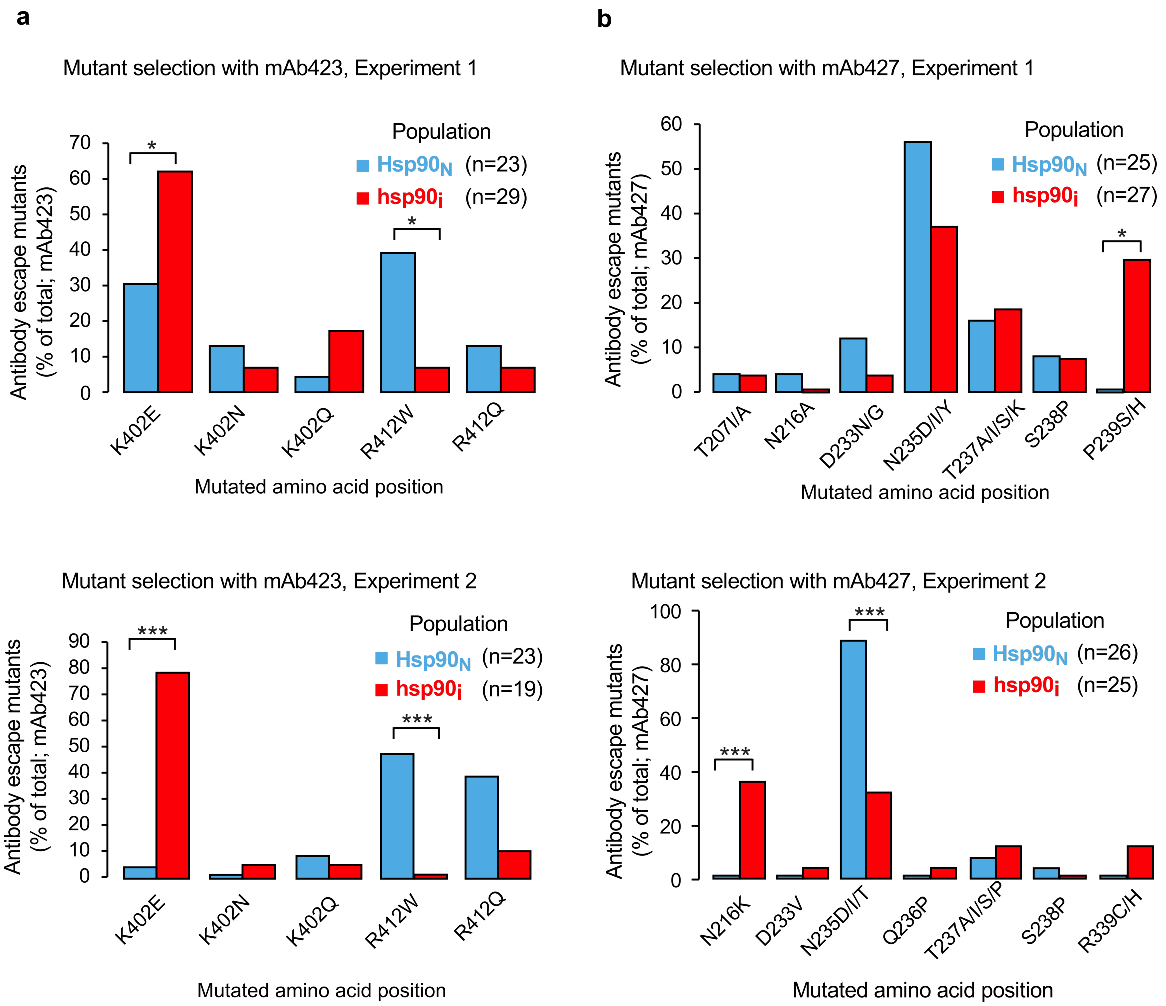

Supplementary Figure 1: Results of individual mutant capsid selection assays.

(**a,b**) Abundance and distribution of mAb423 (a) or mAb427 (b) escape variants in populations of Sabin 1 (a) or wild-type (b) poliovirus from $Hsp90_N$ and $hsp90_i$ populations from 2 separate experiments. The number of mAb-resistant virus variants examined (n) is indicated. Significance: * $p < 0.05$, ** $p < 0.01$, *** $p < 0.005$ by two-tail Fisher's exact test.

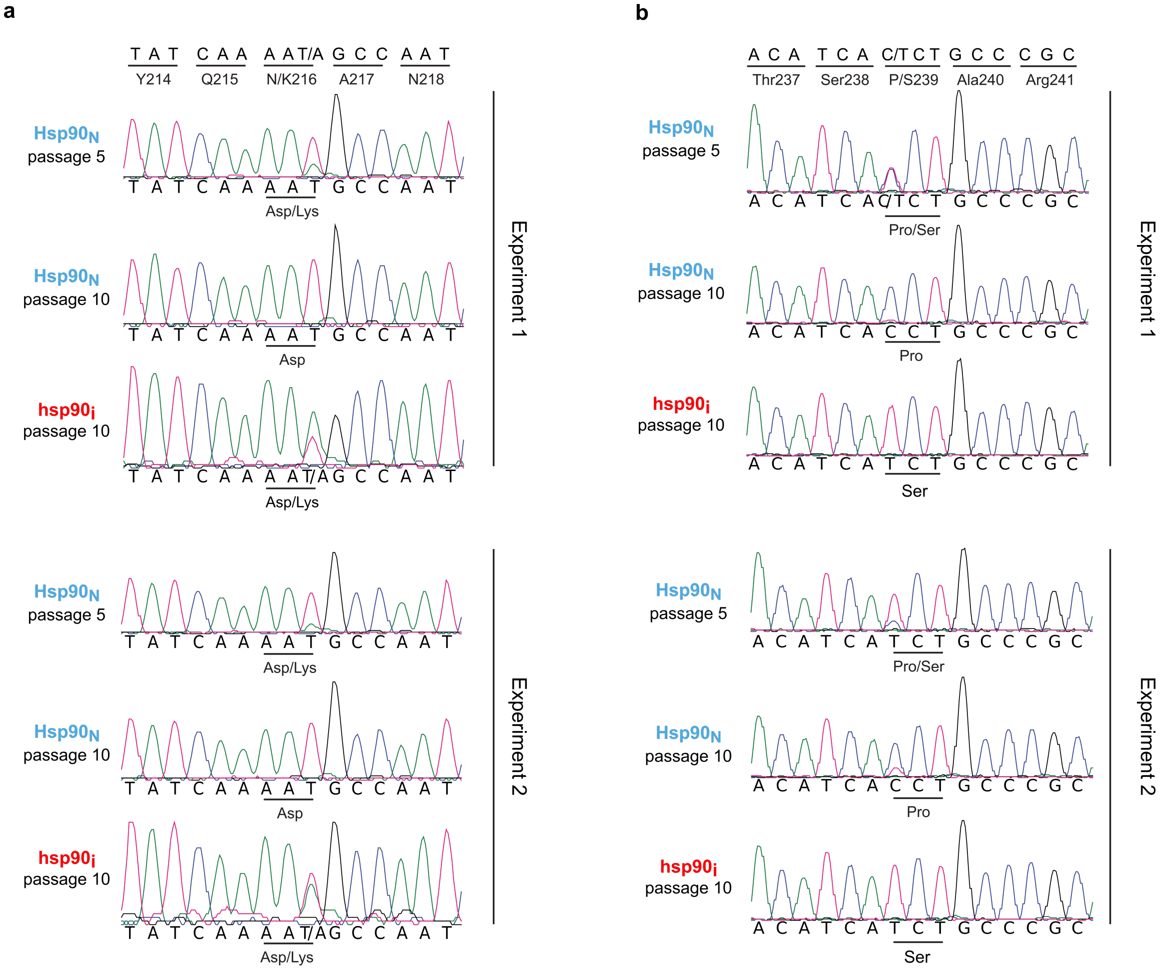

Supplementary Figure 2: Sanger sequencing analysis of competition experiments showing effect of Hsp90 activity on the fitness of escape variants.

(**a**,**b**) Results of competition experiments testing the fitness of N216K (a) or P239S (b) variants under conditions of normal or low Hsp90 activity. An initial infection was performed with a 1:5 ratio of wild-type (N216 or P239) to *hsp90*$_i$ variant (K216 or S239) virus. Viruses were then passaged for a total of 10 passages and competition results assessed by Sanger sequencing the passage 10 virus populations. Intermediate passage 5 results for normal Hsp90 conditions showing mixed population are also shown.

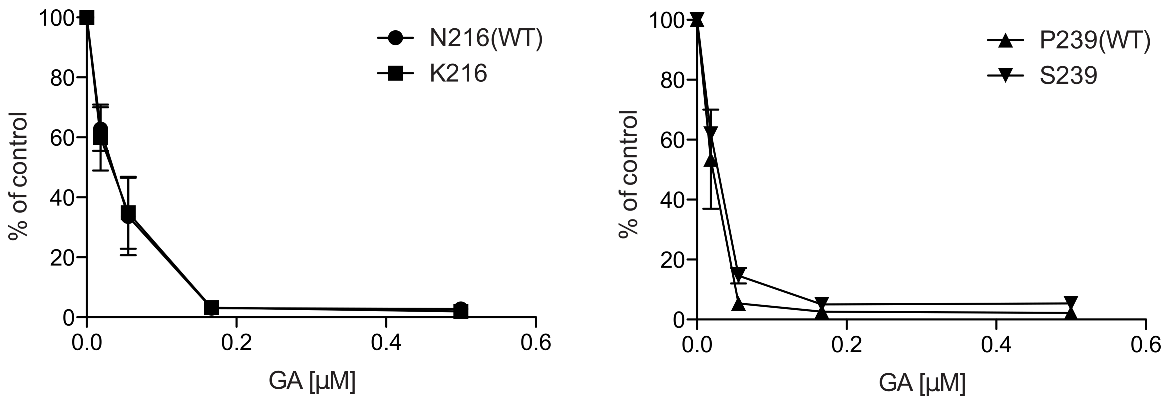

Supplementary Figure 3: mAb escape variants from *hsp90ᵢ* populations do not show reduced sensitivity to Hsp90 inhibition. (**a**) Per cent of virus production relative to no Hsp90 inhibitor (Geldanamycin, GA) for both wild-type and $hsp90_i$ variants K216 and S239 observed to be enriched in mAb selection experiments. Results represent data from 3 experiments, with mean and SEM plotted. No significant differences were observed ($p > 0.1$ by MWW test).

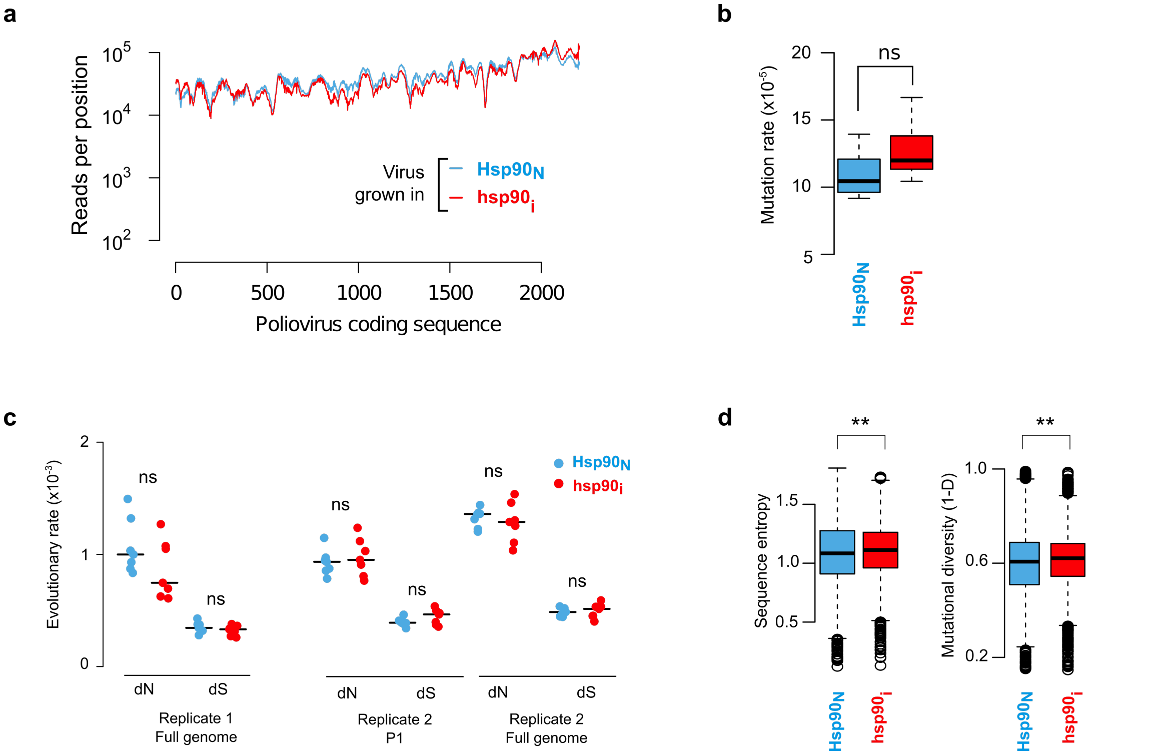

Supplementary Figure 4: Reproducibility of CirSeq analysis

(**a**) CirSeq sequencing coverage of the poliovirus coding region for Replica 2. (**b**) Mutation rate of virus populations in Replica 2. No significance difference is observed for viral passages between $Hsp90_N$ and $hsp90_i$ conditions. (**c**) Evolutionary rates *dN* and *dS* of poliovirus populations. (**d**) Mutational diversity of poliovirus populations for Replica 2. Viral populations passaged under reduced Hsp90 activity ($hsp90_i$) show higher diversity of mutations as estimated by sequence entropy or mutational diversity (1-*D*), with *D* as Simpson's diversity index. For boxplots, the centre line represents the median, the bound box the interquartile range, and the whiskers 1.5x the interquartile range. Significance: ns $p > 0.05$ (not significant), ** $p < 0.01$ by MWW test.

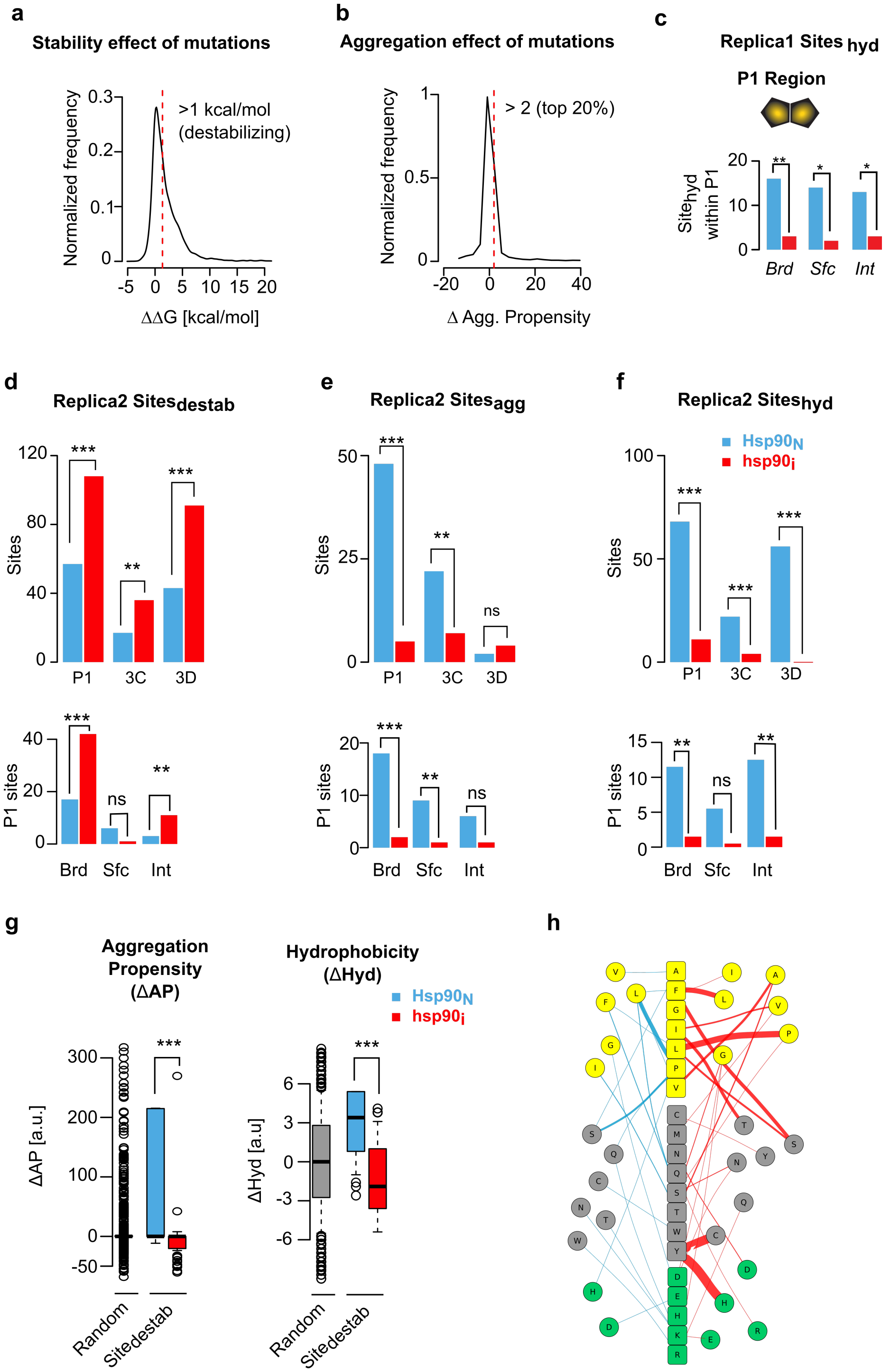

Supplementary Figure 5: Distribution of stability and aggregation effects of variants and reproducibility of variants analyses for replica 2.

(**a**) Distribution of the stability effect of all possible amino acid mutations in poliovirus proteins P1 (capsid), 3C (protease) and 3D (polymerase). Mutations were calculated using the FoldX algorithm and those having a DDG > 1kcal/mol are considered

destabilizing. (**b**) Distribution of the effect of all possible amino acid mutations in poliovirus proteins P1 (capsid), 3C (protease), and 3D (polymerase) on the aggregation propensity predicted with the TANGO algorithm. We classified the top 20%, i.e. mutations that increase the aggregation propensity by more than 2 [a.u.], as aggregation prone mutations. (**c**) The distribution of Site_{hyd} at buried (Brd), interface (Int), or surface (Sfc) positions across P1 based on the capsid crystal structure (PDB: 2PLV) for Replica 1. (**d**) Sites that differ in their ability to accommodate destabilizing variants between $Hsp90_N$ and $hsp90_i$ conditions ($\text{Sites}_{\text{destab}}$) for Replica 2. All analysed viral proteins accommodate more $\text{Sites}_{\text{destab}}$ at reduced Hsp90 activity. Within P1, $\text{Sites}_{\text{destab}}$ differ significantly in buried (Brd) and interface (Int) position, whereas no significant differences were observed for the capsid surface (Srf). (**e**) Sites that differ in their ability to accommodate aggregation prone mutations between $Hsp90_N$ and $hsp90_i$ conditions ($\text{Sites}_{\text{agg}}$) for Replica 2. P1 and 3C accommodate significantly fewer $\text{Sites}_{\text{destab}}$ at lower Hsp90 activity. Within P1, $\text{Sites}_{\text{agg}}$ differ significantly at buried and surface positions. (**f**) Sites that differ in their ability to accommodate hydrophobic mutations between $Hsp90_N$ and $hsp90_i$ conditions ($\text{Sites}_{\text{hyd}}$) for Replica 2. All analysed viral proteins accommodate significantly fewer $\text{Sites}_{\text{hyd}}$ at lower Hsp90 activity. Within P1, $\text{Sites}_{\text{hyd}}$ differ significantly at buried and interface positions across the P1 protein. (**g**) Mutational pleiotropy in Replica 2. Destabilizing mutations enriched in $Hsp90_N$ are significantly more aggregation prone and hydrophobic compared to destabilizing mutations enriched in $hsp90_i$. (**h**) Destabilizing variants observed in $\text{Site}_{\text{destab}}$ in $Hsp90_N$ and $hsp90_i$ populations of replica 2. The edge weight scales with the number of occurrences of variants enriched in $Hsp90_N$ (left) and $hsp90_i$ (right), respectively, and the wild-type amino acid is indicated in the middle. For boxplots, the centre line represents the median, the bound box the interquartile range, and the whiskers 1.5x the interquartile range. Significance: ns $p > 0.05$ (not significant), ** $p < 0.01$, *** $p < 0.001$.

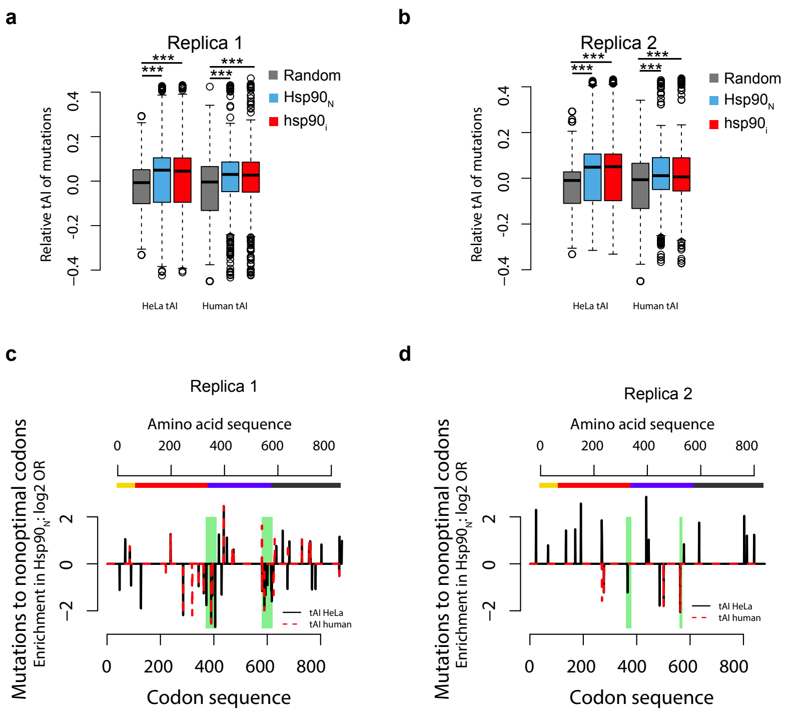

Supplementary Figure 6: Analysis of codon optimality for *Hsp90*$_N$ and *hsp90*$_i$ populations.

(a,b) Change in relative tRNA adaptation index (tAI) of sequence mutations based on either human or HeLa cell tAI for both replica 1 (a) or 2 (b). Under all conditions, codons are in general significantly optimized compared to random mutations. (**c,d**) Sites showing significant codon deoptimization differences between *Hsp90*$_N$ and *hsp90*$_i$ populations in replica 1 (c) or replica (2). For boxplots, the centre line represents the median, the bound box the interquartile range, and the whiskers 1.5x the interquartile range. Significance: *** $p < 0.001$ by MWW test.

Supplementary Table 1: Escape mutants selected with mAb423.
Summary of mAb423 escape mutants from experiments 1 and 2.

Experiment 1

			Nucleotide		Amino Acid		
**Virus Population**	**Clone**	**Position**	**Original**	**New**	**Original**	**New**	**Amino Acid #**
Hsp90$_N$	1	1943	A	G	K	E	402
	2	1943	A	G	K	E	402
	3	1943	A	G	K	E	402
	4	1943	A	G	K	E	402
	5	1943	A	**C**	K	Q	402
	6	1943	A	G	K	E	402
	7	1943	A	G	K	E	402
	8	1943	A	G	K	E	402
	9	1945	A	C	K	N	402
	10	1945	A	T	K	N	402
	11	1945	A	T	K	N	402
	12	1976	C	T	R	W	412
	13	1976	C	T	R	W	412
	14	1976	C	T	R	W	412
	15	1976	C	T	R	W	412
	16	1976	C	T	R	W	412
	17	1976	C	T	R	W	412
	18	1976	C	T	R	W	412
	19	1976	C	T	R	W	412
	20	1976	C	T	R	W	412
	21	1977	G	A	R	Q	412

	22	1977	G	A	R	Q	412
	23	1977	G	A	R	Q	412
hsp90$_i$	1	1943	A	G	K	E	402
	2	1943	A	G	K	E	402
	3	1943	A	G	K	E	402
	4	1943	A	G	K	E	402
	5	1943	A	C	K	Q	402
	6	1943	A	G	K	E	402
	7	1943	A	G	K	E	402
	8	1943	A	G	K	E	402
	9	1943	A	C	K	E	402
	10	1943	A	G	K	E	402
	11	1943	A	C	K	Q	402
	12	1943	A	G	K	E	402
	13	1943	A	g	K	E	402
	14	1943	A	G	K	E	402
	15	1943	A	G	K	E	402
	16	1943	A	G	K	E	402
	17	1943	A	G	K	E	402
	18	1943	A	C	K	Q	402
	19	1943	A	C	K	Q	402
	20	1943	A	G	K	E	402
	21	1943	A	G	K	E	402
	22	1943	A	G	K	E	402
	23	1943	A	C	K	Q	402
	24	1945	A	T	K	N	402
	25	1945	A	T	K	N	402
	26	1976	C	T	R	W	412
	27	1976	C	T	R	W	412

	28	1977	G	A	R	Q	412
	29	1977	G	A	R	Q	412

Experiment 2

			Nucleotide		Amino Acid		
**Virus Population**	**Clone #**	**Position**	**Original**	**New**	**Original**	**New**	**Amino Acid #**
Hsp90N	1	1943	A	C	K	Q	402
	2	1943	A	C	K	Q	402
	3	1943	A	G	K	E	402
	4	1976	C	T	R	W	412
	5	1976	C	**T**	R	W	412
	6	1976	C	T	R	W	412
	7	1976	C	T	R	W	412
	8	1976	C	T	R	W	412
	9	1976	C	T	R	W	412
	10	1976	C	T	R	W	412
	11	1976	C	T	R	W	412
	12	1976	C	T	R	W	412
	13	1976	C	T	R	W	412
	14	1976	C	T	R	W	412
	15	1977	G	A	R	Q	412
	16	1977	G	A	R	Q	412
	17	1977	G	A	R	Q	412
	18	1977	G	A	R	Q	412
	19	1977	G	A	R	Q	412
	20	1977	G	A	R	Q	412

	21	1977	G	A	R	Q	412
	22	1977	G	A	R	Q	412
	23	1977	G	A	R	Q	412
hsp90i	1	1943	A	G	K	E	402
	2	1943	A	G	K	E	402
	3	1943	A	G	K	E	402
	4	1943	A	G	K	E	402
	5	1943	A	G	K	E	402
	6	1943	A	G	K	E	402
	7	1943	A	G	K	E	402
	8	1943	A	G	K	E	402
	9	1943	A	G	K	E	402
	10	1943	A	G	K	E	402
	11	1943	A	G	K	E	402
	12	1943	A	G	K	E	402
	13	1943	A	G	K	E	402
	14	1943	A	G	K	E	402
	15	1943	A	G	K	E	402
	16	1943	A	C	K	Q	402
	17	1945	A	T	K	N	402
	18	1977	G	A	R	Q	412
	19	1977	G	A	R	Q	412

Supplementary Table 2: Escape mutants selected with mAb427.
Summary of mAb427 escape mutants for experiments 1 and 2.

Experiment 1

			Nucleotide		Amino Acid		
**Virus Population**	**Clone #**	**Position**	**Original**	**New**	**Original**	**New**	**AA Number**
Hsp90N	1	1361	A	G	T	A	207
	2	1390	T	A	N	K	216
	3	1439	G	A	D	N	233
	4	1439	G	A	D	N	233
	5	1440	A	G	D	G	233
	6	1445	A	G	N	D	235
	7	1445	A	G	N	D	235
	8	1445	A	G	N	D	235
	9	1445	A	G	N	D	235
	10	1445	A	G	N	D	235
	11	1445	A	G	N	D	235
	12	1445	A	G	N	D	235
	13	1445	A	G	N	D	235
	14	1445	A	G	N	D	235
	15	1445	A	G	N	D	235
	16	1445	A	G	N	D	235
	17	1445	A	G	N	D	235
	18	1446	A	T	N	I	235
	19	1446	A	T	N	I	235
	20	1451	A	G	T	A	237
	21	1451	A	T	T	S	237

	22	1451	A	G	T	A	237
	23	1451	A	G	T	A	237
	24	1454	T	C	S	P	238
	25	1454	T	C	S	P	238
	1	1362	C	T	T	I	207
	2	1440	A	G	D	G	233
	3	1445	G	A	N	D	235
	4	1445	A	G	N	D	235
	5	1445	A	G	N	D	235
	6	1445	A	G	N	D	235
	7	1445	A	G	N	D	235
	8	1445	A	T	N	Y	235
	9	1445	A	G	N	D	235
	10	1445	A	G	N	D	235
	11	1445	A	G	N	D	235
	12	1445	A	G	N	D	235
hsp90i	13	1451	A	G	T	A	237
	14	1451	A	G	T	A	237
	15	1451	A	G	T	A	237
	16	1452	C	T	T	I	237
	17	1452	C	A	T	K	237
	18	1454	T	C	S	P	238
	19	1454	T	C	S	P	238
	20	1457	C	T	P	S	239
	21	1457	C	T	P	S	239
	22	1457	C	T	P	S	239
	23	1457	C	A	P	H	239
	24	1457	C	T	P	S	239
	25	1457	C	T	P	S	239

	26	1457	C	T	P	S	239
	27	1458	C	A	P	H	239

Experiment 2

			Nucleotide		Amino Acid		
**Virus Population**	**Clone #**	**Position**	**Original**	**New**	**Original**	**New**	**AA Number**
Hsp90N	1	1445	A	G	N	D	235
	2	1445	A	G	N	D	235
	3	1445	A	G	N	D	235
	4	1445	A	G	N	D	235
	5	1445	A	G	N	D	235
	6	1445	A	G	N	D	235
	7	1445	A	G	N	D	235
	8	1445	A	G	N	D	235
	9	1445	A	G	N	D	235
	10	1446	A	C	N	T	235
	11	1446	A	C	N	T	235
	12	1446	A	C	N	T	235
	13	1446	A	C	N	T	235
	14	1446	A	C	N	T	235
	15	1446	A	C	N	T	235
	16	1446	A	C	N	T	235
	17	1446	A	C	N	T	235
	18	1446	A	C	N	T	235
	19	1446	A	C	N	T	235
	20	1446	A	C	N	T	235
	21	1446	A	C	N	T	235

	22	1446	A	C	N	T	235
	23	1446	A	T	N	I	235
	24	1451	A	G	T	A	237
	25	1452	C	T	I	I	237
	25	1454	T	C	S	P	238
hsp90i	1	1390	T	A	N	K	216
	2	1390	T	A	N	K	216
	3	1390	T	A	N	K	216
	4	1390	T	A	N	K	216
	5	1390	T	A	N	K	216
	6	1390	T	A	N	K	216
	7	1390	T	A	N	K	216
	8	1390	T	A	N	K	216
	9	1390	T	A	N	K	216
	10	1440	A	T	D	V	233
	11	1445	A	G	N	D	235
	12	1445	A	G	N	D	235
	13	1445	A	G	N	D	235
	14	1445	A	G	N	D	235
	15	1445	A	G	N	D	235
	16	1445	A	G	N	D	235
	17	1445	A	G	N	D	235
	18	1445	A	G	N	D	235
	19	1449	A	C	Q	P	236
	20	1451	A	C	T	P	237
	21	1451	A	G	T	S	237
	22	1451	A	T	T	S	237
	23	1757	C	T	R	C	339
	24	1757	C	T	R	C	339

	25	1758	G	A	R	H	339
